# Carbon amendments in soil microcosms induce uneven response on H_2_ oxidation activity and microbial community composition

**DOI:** 10.1093/femsec/fiad159

**Published:** 2023-12-01

**Authors:** Xavier Baril, Philippe Constant

**Affiliations:** Institut national de la recherche scientifique, Centre Armand-Frappier Santé Biotechnologie, 531 boulevard des Prairies, Laval, Québec H7V 1B7, Canada; Institut national de la recherche scientifique, Centre Armand-Frappier Santé Biotechnologie, 531 boulevard des Prairies, Laval, Québec H7V 1B7, Canada

**Keywords:** carbon, ecological trait, microbial ecology, molecular hydrogen, soil, trace gas

## Abstract

High-affinity H_2_-oxidizing bacteria (HA-HOB) thriving in soil are responsible for the most important sink of atmospheric H_2_. Their activity increases with soil organic carbon content, but the incidence of different carbohydrate fractions on the process has received little attention. Here we tested the hypothesis that carbon amendments impact HA-HOB activity and diversity differentially depending on their recalcitrance and their concentration. Carbon sources (sucrose, starch, cellulose) and application doses (0, 0.1, 1, 3, 5% C_eq_ soil_dw_^−1^) were manipulated in soil microcosms. Only 0.1% C_eq_ soil_dw_^−1^ cellulose treatment stimulated the HA-HOB activity. Sucrose amendments induced the most significant changes, with an abatement of 50% activity at 1% C_eq_ soil_dw_^−1^. This was accompanied with a loss of bacterial and fungal alpha diversity and a reduction of high-affinity group 1 h/5 [NiFe]-hydrogenase gene (*hhyL*) abundance. A quantitative classification framework was elaborated to assign carbon preference traits to 16S rRNA gene, ITS and *hhyL* genotypes. The response was uneven at the taxonomic level, making carbon preference a difficult trait to predict. Overall, the results suggest that HA-HOB activity is more susceptible to be stimulated by low doses of recalcitrant carbon, while labile carbon-rich environment is an unfavorable niche for HA-HOB, inducing catabolic repression of hydrogenase.

## Teaser

Carbon preference traits are not conserved at the taxonomic level in bacteria and fungi but elevated concentrations of labile carbon are deleterious to H2-oxidizing bacteria.

## Introduction

Molecular hydrogen (H_2_) is an indirect greenhouse gas with an average atmospheric mixing ratio of 0.53 ppmv (Novelli et al. 1999). The biological sink of H_2_ is mediated by bacteria possessing high-affinity [NiFe]-hydrogenase, catalyzing the oxidation of H_2_ into two protons and two electrons conveyed to the respiratory chain for ATP generation (Grinter et al. 2023). In general, the energy potential of atmospheric H_2_ is utilized in combination with organic carbon source for mixotrophic survival or growth (Constant et al. 2011, Greening et al. 2014, Liot and Constant 2016). Such diverse trophic capabilities are achieved thanks to the great diversity of H_2_ oxidizing bacteria (HOB) with representatives involved in various biogeochemical cycles (Søndergaard et al. 2016, Greening and Grinter 2022). High-affinity HOB (HA-HOB) are unevenly distributed among different taxonomic groups with a prevalence of Actinomycetota according to metagenomic surveys and characterization of environmental isolates (Constant et al. 2011, Greening et al. 2016). The great diversity and versatility among HA-HOB makes the identification of hallmark conserved traits complicated. Distribution of HOB along organic concentration gradients in desert soil attributed them an oligotrophic life history strategy (Li et al. 2023). This is consistent with the involvement of HA-HOB in the degradation of recalcitrant organic matter in soil (Piché-Choquette and Constant 2019).

Despite the ubiquitous nature of HA-HOB in the environment (Ji et al. 2017, Jordaan et al. 2020, Bay et al. 2021, Lappan et al. 2023), land-use type is a significant driver of the biological sink of atmospheric H_2_, with lower activities in agroecosystems in comparison to forests (Ehhalt and Rohrer 2009). Soil water content, temperature, snow cover, and net primary production were proven efficient to explain spatial and temporal variation of the biological sink of atmospheric H_2_ at the global scale (Hauglustaine and Ehhalt 2002, Ehhalt and Rohrer 2009, Morfopoulos et al. 2012). At the laboratory scale, Khdhiri et al. (2015) proposed a linear model parameterized with soil total carbon content explained the variation of H_2_ uptake rate measured in contrasting soil samples. Other variables including HA-HOB relative abundance, microbial community composition, alpha diversity and pH were correlated with H_2_ oxidation rates (Gödde et al. 2000, Khdhiri et al. 2015, Saavedra-Lavoie et al. 2020, Baril et al. 2022).

The incidence of different carbohydrate fractions on H_2_ oxidation activity and HA-HOB diversity has received little attention. Here, we tested the hypothesis that carbon amendments impact HA-HOB activity and diversity differentially upon their recalcitrance and their concentration in an agricultural soil. The response of HA-HOB was examined along with compositional changes of bacterial and fungal communities to determine whether bacteria engaged in H_2_ oxidation activity have distinctive fate and plasticity in the face of carbon amendment. Soil microcosms were subjected to amendment with sucrose, starch, or cellulose displaying different level of recalcitrance (Kögel-Knabner 2002). The microbial uptake of labile sucrose is mediated by membrane transport protein followed by the catabolism of the disaccharide into fructose and glucose (Reid and Abratt 2005). In contrast, cellulose and starch catabolism require extracellular enzymes, with the former being more recalcitrant than the second due to longer structure (Mizuta et al. 2015). The addition of cellulose was expected to promote the growth and H_2_-oxidation activity because Actinomycetota are prolific producers of cellulases (Berlemont and Martiny 2013) and are among the most abundant HA-HOB in soil (Søndergaard et al. 2016, Ji et al. 2017, Piché-Choquette and Constant 2019). Sucrose was expected to inhibit the process in soil due to carbon repression of hydrogenase activity.

## Materials and methods

### Soil microcosms

The soil for the experiments was collected at INRS, Centre Armand-Frappier Santé Biotechnologie (Laval, Québec, Canada) as described by Agoussar et al. (2021). The soil was sampled in plots located outside ongoing experimental trials. Particle size was homogenized (2 mm sieve) before transferring 10 g soil (dry basis) into 125 ml nominal glass bottles. Each microcosm was amended with either sucrose (Fisher BioReagents FLBP220212), cellulose (Sigma-Aldrich C6429) or starch (J.T.Baker 4006) at different concentrations (0.1, 1, 3, 5%C_eq_ soil_dw_^−1^). C_eq_ refers to the proportion of C atoms added per gram of soil, without considering soil background carbon content. Carbon amendments increased by 0.27 to 13 folds the total carbon content in soil. Soil microcosms with no amendment were used as control. Experiments were designed in three independent blocks (A, B, and C), resulting in 39 microcosms (3 blocks x 3 carbon sources × 4 doses + 1 control repeated three times). A foam plug was inserted to the aperture of microcosms to limit water loss due to evaporation while allowing air exchange during the incubation in the dark at 25°C for 42 days. Soil water content was fixed at 30% water holding capacity. Water loss was compensated on weekly basis by weighting microcosms and adding distilled water. The low water content in soil and the promotion of gaseous exchanges by a large headspace-to-soil volume ratio of 15.7 and a soil layer thickness of 0.5 cm, promoting aerobic conditions. H_2_ oxidation rates were measured weekly during the incubation period with a gas chromatographic assay conducted with an initial H_2_ concentration of 482 ± 142 ppbv (Baril et al. 2022). H_2_ oxidation activity measurements were conducted by integrating the linear decay of H_2_ concentrations in the static headspace of soil microcosms from 5 to 6 observations recorded within less than 20 min. A rate of 0 nmol_H2_ g_dw_^−1^ h^−1^ was assigned to non-significant H_2_ concentration decay (Supplementary materials). Representative soil subsamples were collected in microcosms after 42 incubation days for total DNA extraction and physicochemical analyses. DNA was extracted from soil samples (0.25 g) with the DNeasy PowerLyzer PowerSoil kit (Qiagen®). Total C and N were determined using a Thermo Flash 2000 elemental Analyzer equipped with a thermal conductivity detector (Thermo Fisher Scientific, Pittsburgh, USA). Analyses were performed by the “services des Laboratoires INRS-Centre Eau Terre Environnement (Canada)”. The calibration curve standard was generated with BBOT–2,5-Bis(5-tert-butyl-benzoxazol-2-yl)thiophene (Thermo Fisher Scientific, Pittsburgh, USA). Soil pH (1 g soil suspension in 10 ml 0.01 M CaCl_2_ solution) was measured with an Accumet® model 15 pH-meter (Thermo Fisher Scientific, Pittsburgh, USA).

### Abundance of taxonomic and functional genes

The abundance of bacterial 16S rRNA gene, fungal ITS, *hhyL* gene encoding the large subunit of group 5/1 h NiFehydrogenase were determined by droplet digital PCR (ddPCR) as described in Baril et al. (2022). Briefly, three runs of ddPCR were performed with the ddPCR Gene Expression EvaGreen® Assays (Bio-Rad, Hercules, USA). A random distribution of DNA samples collected was applied and three negative controls with DNA-free sterile water were included. Manual threshold setting was used (Table S2) and fixed to include rain in the positive fraction of the partitions (Huggett 2020). Only samples with more than 10 000 accepted droplets were used for subsequent analyses. Copy number concentrations were converted to gene copy per gram of dry soil.

### Diversity of taxonomic and functional genes

Total genomic DNA was shipped to the *Centre d'expertise et de service Génome Québec* (Montréal, Québec, Canada) for library preparation and PCR amplicon sequencing of bacterial 16S rRNA gene, fungal ITS region, and *hhyL* gene on the Illumina MiSeq PE-250 platform. Primers were the same as those utilized for ddPCR for all genes, but the V4 region of bacterial 16S rRNA gene was PCR-amplified with the primers 515F and 806R (Caporaso et al. 2011). However, *hhyL* PCR amplicons for the 3% and 5% sucrose amendment treatments did not reach the minimum concentration for sequencing and were thus excluded. Raw sequence reads (7650088) were deposited in the Sequence Read Archive of the National Center for Biotechnology Information under Bioproject PRJNA1015275. Primer sequences were removed from raw reads with cutadapt (Martin 2011). Downstream sequence quality control, amplicon sequence variant (ASV) denoising, and taxonomic assignation was done with the R package “dada2” version 1.16.0 with default parameters (Callahan et al. 2016). For the three genes, a minimum threshold of 0.005% relative abundance was attributed to reduce noise in ASV tables (Bokulich et al. 2013). Following this cut-off, 1503 (1322438 reads), 293 (1919561 reads) and 648 (426297 reads) ASV were kept for 16S rRNA gene, ITS and *hhyL* analyses, respectively. The taxonomic assignation of 16S rRNA gene and ITS region was based on SILVA version 138 (Quast et al. 2012) and UNITE database version 7.2 (UNITE Community 2017) databases, respectively. Taxonomic assignation of *hhyL* gene was not undertaken due to frequent lateral transfer events (Constant et al. 2011). Alpha diversity of each gene was expressed with the extrapolated value of the first three Hill numbers representing species richness (q = 0), the exponential function of the Shannon entropy index (q = 1), and the inverse of Simpson index (q = 2). For each microcosm, alpha diversity values were extrapolated with the package “iNEXT” version 2.0.20 (Chao et al. 2014).

### Statistical analysis

Statistical analyses were performed using the software R version 4.1.2 (R Core Team 2021). The variation in H_2_ oxidation rate over the 6 weeks monitoring period was examined with simple linear regression on Ln-transformed data with the package “stats” version 4.1.2 (R Core Team 2021). The median of H_2_ oxidation rates measured in each microcosm (8 observations over 42 days) was utilized to examine the relation between process rates and soil physicochemical properties as well as microbial community. The effect of carbon amendment treatments (carbon sources and doses), on median H_2_ oxidation rate, microbial communities (gene copy number and alpha diversity) and soil physicochemical properties (pH, total carbon and total nitrogen) was examined with two-way ANOVA followed by a Tukey *post hoc* test using the package “stats” version 4.1.2 (R Core Team 2021). The potential block effect on these variables was tested with one-way ANOVA. Spearman correlation between H_2_ oxidation rate and bacteria, fungi, HA-HOB abundance and alpha diversity were verified with the package “corrplot” version 0.92 (Wei and Simko 2021). The effect of amendment treatments on beta diversity of soil microbial communities was tested with a Permutational multivariate analysis of variance (PERMANOVA) on the Bray–Curtis distance matrix computed on Hellinger-transformed read counts, generated with the package “vegan” version 2.6.4 (Anderson 2014, Oksanen et al. 2020). Multilevel pairwise comparisons were computed with the package “pairwiseAdonis” version 0.4.1 (Arbizu 2017). Principal Coordinates Analysis (PCoA) was executed with the package “phyloseq” version 1.38.0 (McMurdie and Holmes 2013) and graphical outputs were generated with the package “ggplot2” version 3.3.6 (Wickham 2016). The level of rejection was set at α = 0.05 for all statistical tests.

### Assignation of carbon preference traits

Assignation of carbon preference traits (C-traits) was undertaken at the ASV level for the three PCR amplicons utilizing data from soil microcosms exposed to 1% C_eq_ sucrose, starch and cellulose. This concentration was chosen owing to the abatement of the H_2_ oxidation activity by 50% compared to control in soil amended with sucrose. The classification framework comprises three axes, named C-ness, St-ness, and Su-ness defining the prevalence of each ASV as a function of cellulose, starch and sucrose soil amendments, respectively. ASV with less than 3 non-zero observations out of 9 samples were removed from the ASV tables to limit structural zero. Bias regarding library size between treatments was verified with Kruskal-Wallis test (*P* > 0.11). Centered log-ratio (clr) transformation was applied on the ASV tables to address the closed nature of the sequencing data (Quinn et al. 2018) and a pseudocount representing the absolute value of the smallest log-ratio was added to the transformed data to enable the computation of relative abundance of ASV tables. Carbon preference traits were assigned according to the three axes of the classification framework. Those axes were obtained by dividing the average of the three replicates of an ASV clr value in a treatment by the sum of the ASV clr average values computed in the three treatments. For instance, the C‐ness of ASVx in soil amended with carbon *i* (C-ness_(x, i)_) is computed with the following equation:


(1) \begin{eqnarray*}
C{\text -}nes{s}_{\left( {x,i} \right)} = \frac{{{{\bar{x}}}_i}}{{{{\bar{x}}}_i + {{\bar{x}}}_j + {{\bar{x}}}_k}}
\end{eqnarray*}

Where ${\mathrm{\bar{x}}}$ corresponds to the average clr value of ASVx observed in the biological replicates, and the subscripts *i, j*, and *k* denote cellulose, starch and sucrose treatments, respectively. The value obtained for each trait were comprised between 0 and 1. The equation was applied for each ASV and each treatment (C-ness, St-ness, and Su-ness) to position ASV along the three axes of a ternary plot. The significance of the assigned traits was tested by comparing change in ASV log-ratio between 1% amendment and control with Analysis of Compositions of Microbiomes with Bias Correction (ANCOM-BC2) computed with the package “ANCOMBC” version 1.6.3 (Lin and Peddada 2020). R scripts for the assignation of carbon preference traits are available on the GitHub project at https://github.com/xbaril/R_script_C-traits.

## Results

### H_2_ oxidation rates

**Figure 1.**
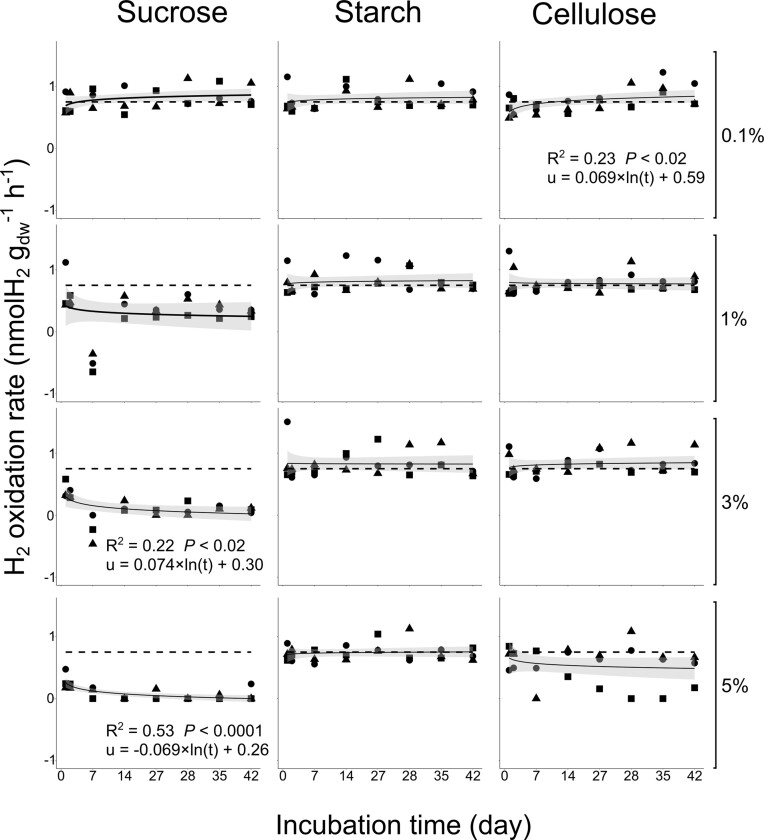
H_2_ oxidation rate (*u*) for the incubation period (*t*). Dash line represent H_2_ oxidation rates measured in the control, and solid line represent logarithmic model. Negative values indicate net emissions of H_2_. Regression equations are only shown for significative models. Circle, triangle and square represent the A, B, and C replicates, respectively.

H_2_ oxidation rate was measured weekly for 42 days to monitor change in activity in response to carbon amendment in soil (Fig. 1). In general, measurements showed a net uptake of H_2_, whereas the absence of a significant uptake or even net emission were observed at certain measurement points for sucrose amendments. The time series of the activity was in a steady state in control (*P* > 0.5) and starch (*P* > 0.4) microcosms. The sole treatment that triggered a rise of H_2_ oxidation rate during the incubation was 0.1% C_eq_ soil_dw_^−1^ cellulose, whereas microcosms with the two highest doses of sucrose (3 and 5%) followed the opposite trend (Fig. 1). Distinct temporal series noticed among treatments was supported by a comparative analysis of median H_2_ oxidation rates measured in microcosms during the whole incubation. Carbon sources, carbon concentrations, and their interaction explained variation of H_2_ oxidation rate (two-way ANOVA, *P* < 0.0001), without experimental block effect (one-way ANOVA, *P* > 0.6). Sucrose amendments had the greatest impact on oxidation rate, with a loss of activity proportional of the sucrose dose (Table 1). Median H_2_ oxidation rate was significantly different between control and microcosms amended with 1, 3 and 5% C_eq_ soil_dw_^−1^ sucrose (Tukey, *P* < 0.002). Under these conditions, up to 92% of the H_2_ oxidation activity was loss.

**Table 1.** Median H_2_ oxidation rate and soil biotic and abiotic properties for each treatment.

Carbon type	Amendment doses (%)	H_2_ (*u*) median	pH	C:N	Bacteria abundance^a^	Fungi abundance^a^	HA-HOB abundance^a^	16S rRNA gene SR	ITS SR	*hhyL* SR
Control	0	0.75 ± 0.07	6.68 ± 0.08	11.9 ± 0.6	5.6 ± 4.0	0.09 ± 0.02	0.82 ± 0.36	718 ± 98	174 ± 9	158 ± 15
Starch	0.1	0.74 ± 0.09	6.72 ± 0.08	12.0 ± 0.5	3.9 ± 2.4	0.15 ± 0.19	0.85 ± 0.66	639 ± 29	169 ± 16	139 ± 19
	1	0.74 ± 0.01	6.79 ± 0.22	16.9 ± 1.7	2.4 ± 1.9	0.49 ± 0.17	0.96 ± 1.11	611 ± 153	97 ± 40	158 ± 4
	3	0.75 ± 0.05	6.82 ± 0.20	23.4 ± 1.4	2.2 ± 1.3	0.46 ± 0.12	0.61 ± 0.28	718 ± 62	109 ± 21	147 ± 14
	5	0.71 ± 0.02	6.70 ± 0.07	39.7 ± 4.7	3.7 ± 3.7	0.40 ± 0.12	0.69 ± 0.64	569 ± 219	101 ± 53	135 ± 27
Cellulose	0.1	0.71 ± 0.08	6.61 ± 0.22	12.2 ± 1.2	3.6 ± 3.9	0.15 ± 0.11	1.13 ± 1.41	716 ± 58	173 ± 5	156 ± 13
	1	0.74 ± 0.06	6.75 ± 0.08	15.7 ± 0.9	4.5 ± 0.6	0.47 ± 0.18	0.87 ± 0.31	725 ± 52	162 ± 8	147 ± 10
	3	0.80 ± 0.09	6.71 ± 0.13	26.1 ± 2.4	3.6 ± 3.2	0.64 ± 0.31	0.93 ± 0.47	815 ± 173	156 ± 27	70 ± 62
	5	0.52 ± 0.23	6.71 ± 0.09	22.1 ± 9.6	4.7 ± 2.6	1.25 ± 1.09	1.26 ± 0.58	674 ± 116	122 ± 20	133 ± 11
Sucrose	0.1	0.75 ± 0.07	6.74 ± 0.18	12.3 ± 0.9	5.4 ± 3.2	0.09 ± 0.05	0.98 ± 0.87	736 ± 73	172 ± 12	150 ± 10
	1	0.36 ± 0.10	7.20 ± 0.28	11.8 ± 0.3	2.9 ± 1.4	0.28 ± 0.10	0.20 ± 0.12	429 ± 86	105 ± 12	98 ± 17
	3	0.10 ± 0.01	7.52 ± 0.47	14.3 ± 0.4	3.4 ± 1.7	1.10 ± 0.17	0.06 ± 0.04	187 ± 50	52 ± 10	NA
	5	0.06 ± 0.05	7.10 ± 0.29	16.0 ± 0.3	2.1 ± 2.8	1.83 ± 0.23	0.04 ± 0.03	187 ± 99	24 ± 2	NA

Error margins represent standard deviations from the three replicates.

*u*: H_2_ oxidation rate in nmol_H2_ g_dw_^−1^ h^−1^.

a : gene copies × 10^8^ per gram of soil_dw_

SR: Species richness

### Soil microbial communities

**Figure 2.**
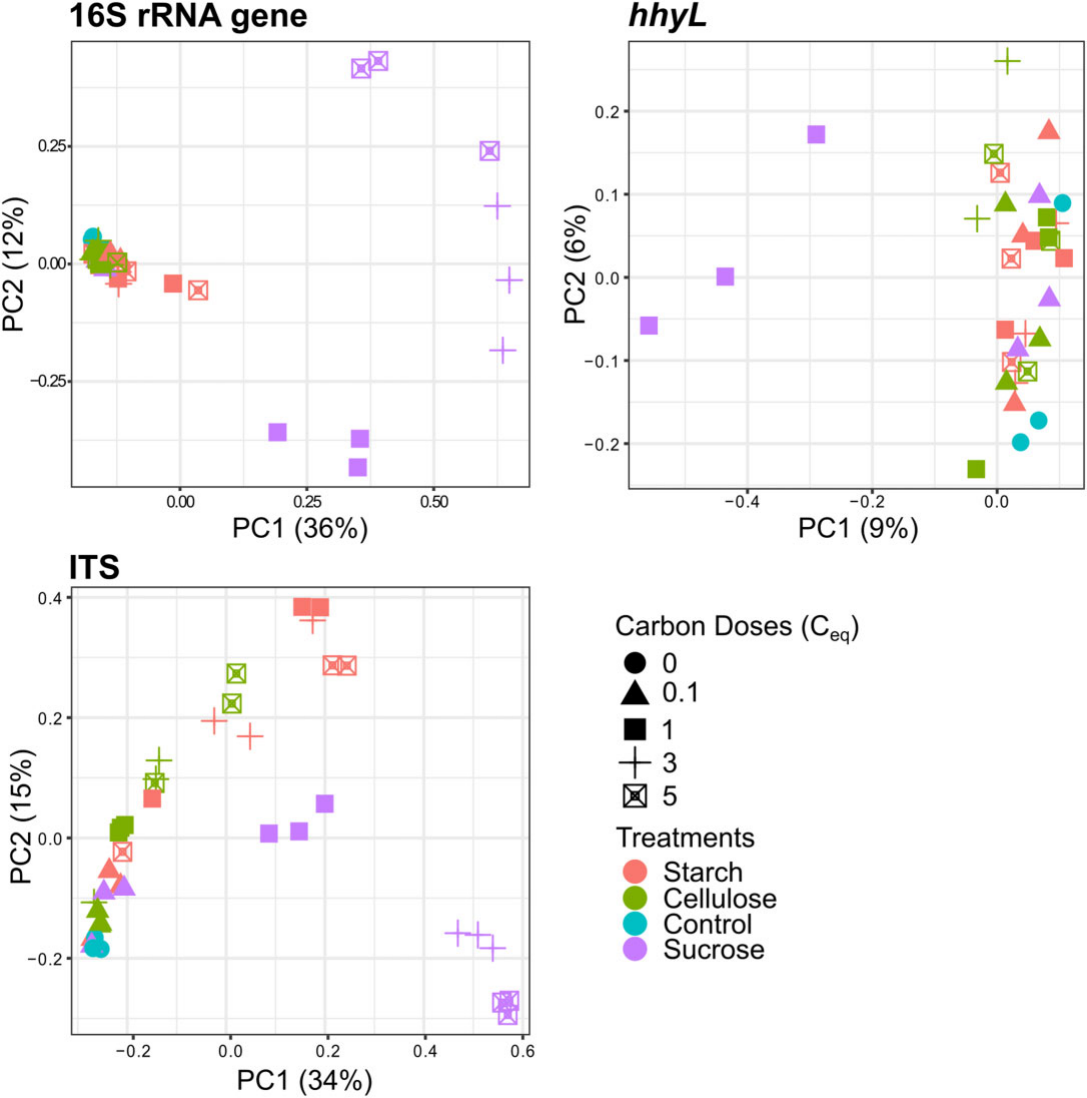
Principal Coordinates Analysis (PCoA) for bacterial, fungal and HA-HOB communities in soil amended with different doses of sucrose, starch, or cellulose.

Diversity and abundance of bacterial 16S rRNA gene, fungal ITS and HA-HOB *hhyL* gene were analyzed to disentangle their contribution in explaining contrasting H_2_ uptake activities among soil microcosms. The variation of the three alpha diversity metrics was explained by amendments treatments and doses as well as their interaction for bacteria (two-way ANOVA, *P* < 0.0004) and fungi (two-way ANOVA, *P* < 0.03), whereas only the species richness of *hhyL* followed the same pattern (two-way ANOVA, *P* < 0.011). Sucrose treatments were the main driver of these patterns, reducing the alpha diversity of bacteria and fungi. These reductions of microbial diversity were correlated with the loss of H_2_ oxidation activity triggered by sucrose amendments (Fig. S2). For fungi, the loss of species richness was paralleled with a higher abundance of ITS gene copy number (ρ < − 0.64, *P* < 0.0001), indicating the enrichment of fungal species under elevated sucrose concentrations. In contrast, neither the abundance of bacteria (two-way ANOVA, *P* > 0.4) nor the abundance of HA-HOB (two-way ANOVA, *P* > 0.06) was significatively related to amendment type or doses. The abundance of HA-HOB however declined with sucrose dose, with a one-log scale decrease from the 3% C_eq_ soil_dw_^−1^ dose onwards when compared to control (Table 1). The composition of microbial communities was dominated by ASV encompassing the bacterial classes Actinomycetes (16%), Thermoleophilia (16%) and Alphaproteobacteria (15%), whereas fungi were essentially dominated by Sordariomycetes (67%). The interaction between carbon type and dose explained variation of bacteria, fungi, and HA-HOB community structure (Table 2). The pattern was mainly driven by sucrose treatments (Fig. 2). The composition of fungal and bacterial communities was distinguishable to a lesser extent between starch and cellulose treatments (PERMANOVA, R^2^ > 0.07, *P-adj* < 0.04). Carbon sources, carbon concentrations, and their interaction explained total C accumulation and increase in C: N ratio in soil (two-way ANOVA, *P* < 0.0001). The increase in C: N ratio was observed in cellulose and starch treatments (Tukey, *P* < 0.009) due to their lower mineralisation than sucrose.

**Table 2.** Incidence of carbon sources and their doses on the composition of bacterial, fungal and HA-HOB communities. The contribution of each factor and their interactions was examined with a PERMANOVA.

Gene	Variable	R^2^	*P-value*
16S	Carbon type	0.30	0.001
	Carbon dose	0.07	0.001
	Dose × Type	0.13	0.001
ITS	Carbon type	0.34	0.001
	Carbon dose	0.19	0.001
	Dose × Type	0.14	0.001
*hhyL*	Carbon type	0.15	0.002
	Carbon dose	–	n.s.
	Dose × Type	0.13	0.001

PERMANOVA results for *hhyL* do not include the 3 and 5% C_eq_ soil_dw_^−1^ sucrose treatments, as there was no amplification of this gene in these treatments.

### Carbon preference traits

**Figure 3.**
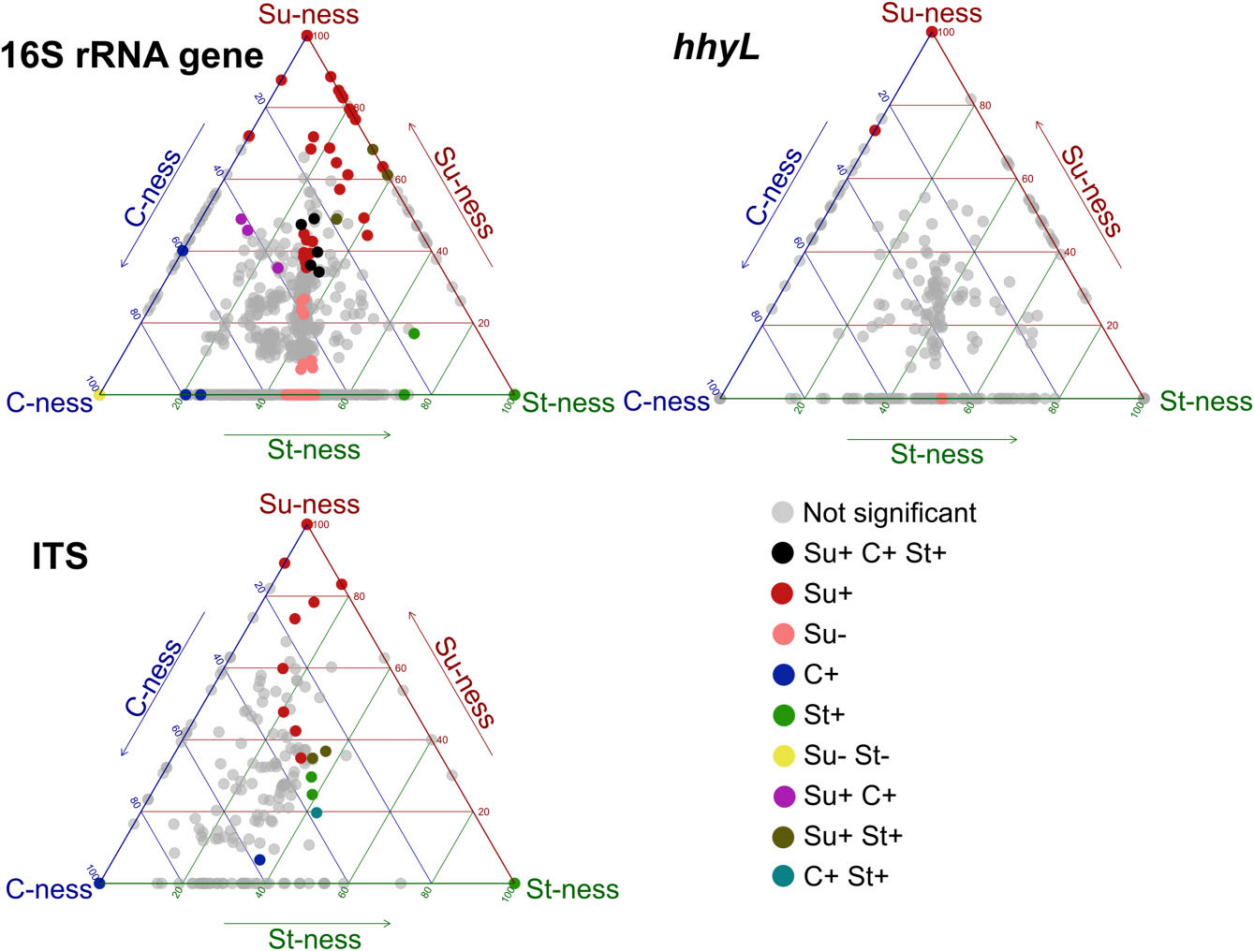
Assignation of C-trait for bacteria, fungi and HA-HOB. Point position in the plot refers to the ASV relative log-ratio value in the 1% treatments of sucrose, cellulose and starch, respectively. Significance of the assignation in the classification scheme was established with ANCOM-BC test. Light grey points were not significantly impacted by treatments compared to the control. Black point are ASV that were positively affected by all carbon amendments. Colored point were significantly impacted by the specified treatment compared to the control. Negatively impacted ASV in all treatment (Su-, C-, St-) were not included in the plot.

**Figure 4.**
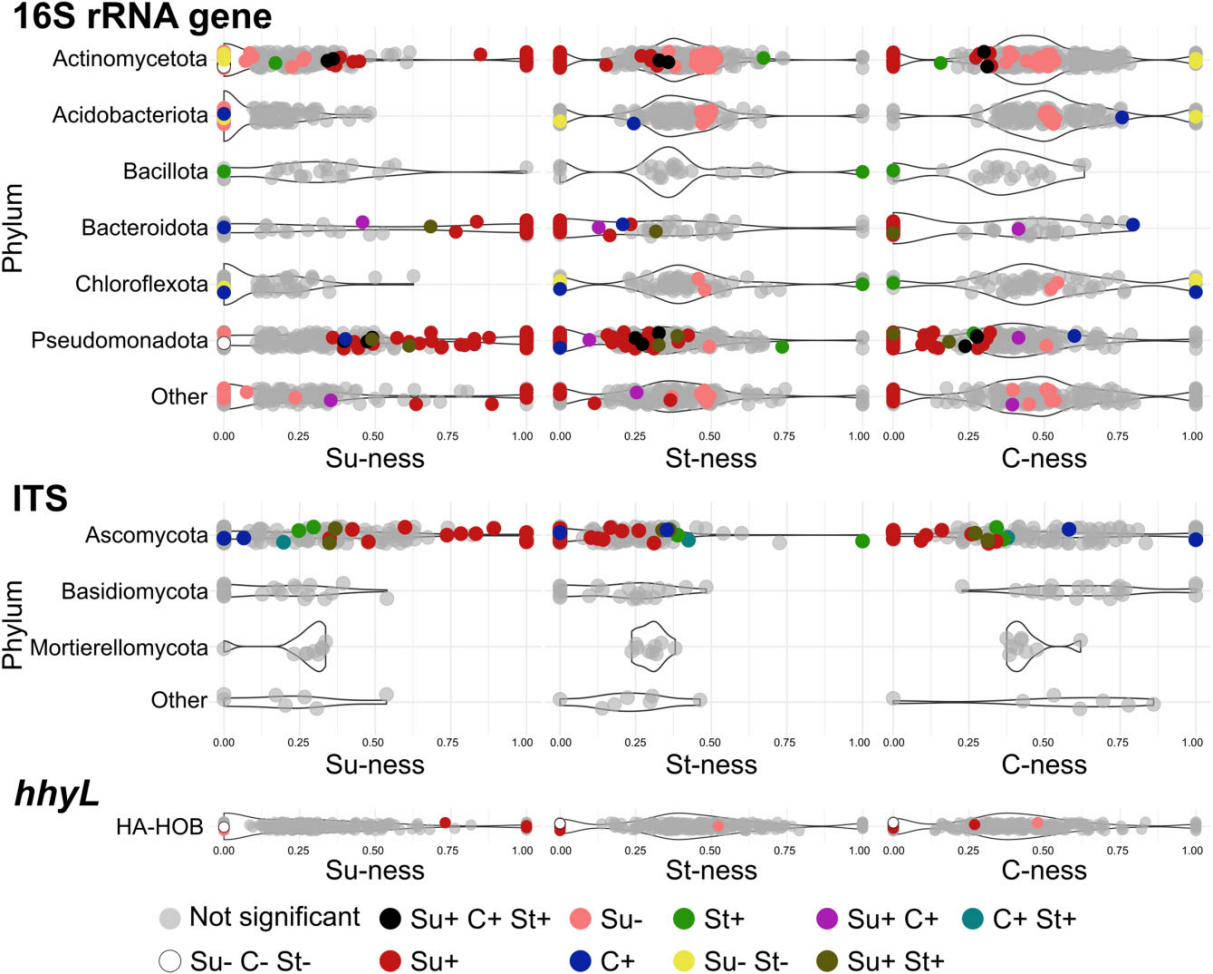
Distribution of C-traits of bacterial and fungal ASV classified at the phylum level, and HA-HOB ASV.

C-traits of ASV were determined based on their relative abundance across different carbon amendment treatments (Fig. 3). Despite their abatement in larger sucrose doses, HA-HOB were less responding to C amendments (6 ASV; 1%) than fungi (22 ASV; 8%) and bacteria (173 ASV; 12%). Bacteria ASV responding significantly to treatment based on Su-ness trait, either positively or negatively, accounted for 95% (165/173). Among them, 113 ASV were positively impacted by sucrose, encompassing different phyla such as Pseudomonadota (66), Actinomycetota (20) and Bacteroidota (20). Five bacterial ASV were positively impacted by the addition of all three carbon sources, which were associated with phyla Pseudomonadota (3) and Actinomycetota (2). In contrast, the three carbon amendments had a negative impact on two Actinomycetota and one Pseudomonadota representative. In total, 52 bacterial ASV were negatively impacted by sucrose amendment with a prevalence of Actinomycetota (26) and Acidobacteriota (10). Moreover, fungal community traits show a continuum including both extreme and intermediate positions along Su-ness and C-ness axes. In total, 22 fungal ASV were significatively affected by treatment, almost all (95%) representant of the Ascomycota phylum (Fig. 4). Sucrose was the main treatment responsible for those changes in fungal ASV abundances (68%). Finally, HA-HOB C-traits were aligned along a continuum encompassing extreme and intermediate position along the three axes of the classification scheme (Fig. 4). Most significant responses of HA-HOB were related to Su-ness. Of these, two and four were negatively and positively impacted by sucrose, respectively. Significant *hhyL* ASV sequences were aligned against the NCBI type material database with the Basic Local Alignment Search Tool (BLAST) to identify their closest relatives. Four of the six significant ASV were closely related to NiFe-hydrogenase genes found in Actinomycetota.

## Discussion

### Direct and indirect effects of carbon amendments on H_2_ oxidation activity

The addition of carbon in high amounts in soil microcosms is expected to have triggered a priming effect leading to enhanced degradation of the soil organic matter pool. The intensity of that priming effect varied among the different carbon sources depending on their recalcitrance and their bioavailability determined by their solubility (Blagodatskaya et al. 2009). Effects of carbon amendments on fermentation, on catabolic repression of hydrogenase and on microbial interactions are proposed to explain the variation of H_2_ oxidation activity measured in soil microcosms.

H_2_ evolution was higher than H_2_ oxidation rate after one week in the 1 and 3% sucrose treatments, resulting in a net emission of H_2_ (Fig. 1). While O_2_ diffusion was promoted in soil microcosms, rapid sucrose-induced respiration may have resulted in anaerobic conditions within small (< 2 mm) soil aggregates, leading to the production of H_2_ by dark fermentation (Dunbar et al. 2023). The transient increase in H_2_ production in the soil is typically offset by a concomitant increase in HOB activity (Piché-Choquette et al. 2018); this is the case for the high amount of H_2_ emitted by nitrogen-fixing nodules, which is consumed in the first few centimeters surrounding the nodule (La Favre and Focht 1983). The net H_2_ production during the first week of incubation did not result in a sufficient stimulation of the H_2_ uptake rate to compensate. A first explanation of the decreasing trend in net uptake of H_2_ with high doses of sucrose therefore is an elevation of the compensation point (H_2_ concentration in the static headspace when both oxidation and production processes are in equilibrium) caused by fermentation metabolism (Conrad 1994).

As a labile source of carbon, sucrose was the most influential on microbial community composition and H_2_-oxidation activity. The disaccharide is readily available to microorganisms through permeases, phosphotransferase and ABC transporter systems channeling the sugar to subsequent glycolysis metabolism (Reid and Abratt 2005). Energy generation changes the energy status in the cell, leading to pleiotropic alteration of gene expression, including an abatement of hydrogenase activity (Friedrich et al. 1981, Eberz and Friedrich 1991). Such a catabolic repression has been observed in various HOB isolates, including the model *Ralstonia eutropha* H16 switching between lithoautrotrophic and heterotrophic growth (Schwartz et al. 2009) and the HA-HOB *Mycobacterium smegmatis* using a mixotrophic growth strategy (Berney and Cook 2010, Greening et al. 2014). In *Streptomyces* spp., hydrogenase activity is expressed in spores generated for long-term persistence and facilitating dissemination (Constant et al. 2010). Catabolic repression of hydrogenase activity in HA-HOB affiliated to *Streptomyces* can be related to spore germination or sporulation inhibition following the increased availability of labile carbon (Ensign 1978). H_2_ oxidation activities measured in soil microcosms potentially involve various HA-HOB encompassing Actinomycetota, Pseudomonadota, Chloroflexota, Bacteroidota, and Acidobacteriota (Søndergaard et al. 2016, Piché-Choquette and Constant 2019). The relative contribution of each phylum to the biological sink of H_2_ remains to be determined, but carbon repression is likely a conserved response among HA-HOB displaying mixotrophic metabolism. This response was unique to sucrose amendments, the most labile carbon source integrated in soil microcosms. As recalcitrant carbohydrates, cellulose, and starch exerted less incidence on microbial communities and H_2_ oxidation activity. The metabolism of theses carbon sources requires extracellular enzymes, hydrolysing polymers in reduced sugar translocated into the cell by dedicated transport systems. On the basis of the saprophyte metabolism of HA-HOB in soil (Piché-Choquette and Constant 2019), starch and cellulose were expected to promote H_2_ oxidation activity. The process was only stimulated by 0.1% cellulose amendment and did not differ from the control after other starch or cellulose treatments (Fig. 1). Nitrogen limitation in soil amended with carbon may account for the lack of stimulation of H_2_ uptake.

The presumed catabolic repression of H_2_ oxidation activity induced by sucrose paralleled with the establishment of a suitable niche for copiotrophic microorganisms. Such a transition in ecological traits was reported in a previous work demonstrating the enrichment of fast growing organisms (r-strategists) to the detriment of K-strategists in glucose-amended soils (Blagodatskaya et al. 2009). The proportion of ASV responding to the treatment was higher for bacteria and fungi than for HA-HOB genotypes. Unsuccessful PCR amplification of *hhyL* gene for sequencing library preparation and the reduction in *hhyL* gene copy number in the 3% and 5% C_eq_ soil_dw_^−1^ sucrose treatments indicate a less favorable niche for HA-HOB. This was also observed in extreme oligotrophic environment where labile carbon amendment favored copiotrophic traits at the expense of HA-HOB activity (Li et al. 2023). Community shifts favoring competitive traits with labile carbon availability could also trigger antagonistic interactions with HA-HOB or disrupt beneficial interactions (Wood et al. 2023). This interpretation is compatible with the decline of bacterial and fungal alpha diversity correlated with loss of H_2_ oxidation activity in soil microcosms (Fig. S2). The role of microbial interaction on the distribution and activity of trace gas oxidizers has however received little attention. Supporting experimental evidence has mostly been obtained for methanotrophic bacteria. The assembly of synthetic communities led to the observation that enhancement of species richness promotes the CH_4_ oxidation activity of *Methylomonas methanica* NCIMB 11130^T^ (Ho et al. 2014). More mechanistic insights were reported in the case study of *Pseudomonas mandelii* producing volatile compounds exerting either positive or negative effects on the activity of methanotrophic bacteria (Veraart et al. 2018). At the community level, distribution of methanotrophic bacteria was partly constrained by other members of the microbial community in water (Guggenheim et al. 2020). There is no similar study for HA-HOB, impairing mechanistic insights driving their incompatibility with copiotrophic ecological niches.

### Assignation of C-trait

The method used in this work allowed the assignation of C-traits to bacteria, fungi, and HA-HOB at the ASV level. A challenge experienced in the classification scheme was the inherent problem of the closed nature of the sequencing data (Quinn et al. 2018). Coordinates of the ASV inside the framework were calculated on the basis of transformed relative abundance, leading to potential interpretation bias toward alteration of absolute abundance of genotypes. Position of ASV along the three axis is therefore a qualitative assignment that must be taken carefully. The application of ANCOM-BC integration estimation of absolute abundance allowed to test the significance of distribution patterns of ASV in microcosms amended with carbon compared to the control. This approach enabled a more stringent assignation of carbon preference traits to a few ASV (from 1% to 12%). The centered position of the triple-positive ASV for cellulose, starch, and sucrose within the framework shows agreement between the ANCOM-BC results and the framework computation result (Fig. 3). The method presented in this study was assessed using specific C-traits as part of a case study for a single soil. Application of the method in more soils and as a framework for examining other pertinent traits related to HA-HOB, (e.g. affinity for H_2_) is expected to improve predictability of H_2_ oxidation process in response to environmental changes.

Classifying organisms according to their traits, or life strategies, has long been a goal for ecologist. The work of Ho et al. (2013) was pioneering by attributing the three life strategies developed for plants to methanotrophic bacteria on the basis of their distribution in various environments. The ecological traits of HA-HOB are not extensively documented. Therefore, observed bacterial and fungal C-traits were employed as a reference for defining the sensitivity of HA-HOB related to the other members of soil microbial communities. The elaboration of a C-trait classification framework was not achieved at the taxonomic level. Despite the loss of H_2_ oxidation activity with sucrose, the most responsive HA-HOB ASV were predominantly favored by the sugar. Such a decoupling between traits and process rates also applies C-trait at the taxonomic level. Nearly all phyla were represented by ASV showing C-trait encompassing extremes and intermediate positions in the classification scheme. Conserved C-traits at the phylum level was only observed for Acidobacteriodota and Basidiomycota that responded negatively to sucrose. This response is in line with their assignment of oligotrophic life strategies (Fierer et al. 2007, Yao et al. 2017). Nevertheless, the high level of variation of Su-ness of ASV encompassing Actinomycetota and Pseudomonodota reiterates the difficulty of generalizing ecological traits to the level of the entire phylum (Stone et al. 2023). The idiosyncratic responses of microorganisms to carbon amendments observed here, combined with the observation of higher species-specific variations of quantitative traits within a guild when compared to inter-guild (Westoby et al. 2021) question the relevance of taxonomic or functional classification system to predict ecological traits of microorganisms. Flexible metabolism and adaptation capabilities of microorganisms impair generalizations in trait attribution. This implies that simplified models assuming monotonous response of HA-HOB to different carbon reservoir components (Li et al. 2023) must be taken with caution. Approaches directed to the genotype level (e.g. ASV, MAG) would be more efficient to examine adequation between ecological traits and distribution of microorganisms in contrasting environmental conditions. Inclusion of indirect effects triggered by microbial interactions governing the activity of HA-HOB also poses a significant challenge. Future ecological trait classification frameworks integrating abiotic and biotic features of extensive soil survey in machine learning environments would definitely contribute to address such a limitation.

## Conclusion

This case study aimed to validate the effect of recalcitrant and labile carbon amendment on HA-HOB activity, community structure, and abundance. The activity was lightly stimulated by low doses of more recalcitrant carbon and more markedly inhibited by high doses of labile carbon. The abatement of activity was accompanied by a decrease in *hhyL* gene copy number and richness. Bacteria and fungi communities showed pattern in their response to the treatments, but these were uneven across taxa. The incorporation of multiple traits is the next step to decipher life history strategies and understand the variables governing the activity of HA-HOB in soil. Integration of more complex substrates such as compost and crop residues in the C-trait classification framework and exploration of nitrogen limitation on H_2_ oxidation activity is also recommended for future investigations.

## Supplementary Material

fiad159_Supplemental_Files
