# Supplementary material for: Carbon amendments in soil microcosms induce uneven response on H2 oxidation activity and microbial community composition
Source: FEMS Microbiol Ecol. 2023 Dec 1;99(12):fiad159. doi: 10.1093/femsec/fiad159 (PMC10716739; doi:10.1093/femsec/fiad159)
Supplement: fiad159_Supplemental_Files [file fiad159_supplemental_files.zip › Supplementary_Data Method.docx]

Table S1. Cycling condition for Bio-Rad’s C1000 Touch Thermal Cycler used for the assays.

| Temperature (°C) | Time | Number of Cycles |
| --- | --- | --- |
| 95 | 5 min | 1 |
| 95 | 30 sec | 50 |
| Varying | 1 min |  |
| 72 | 30 sec |  |
| 4 | 5 min | 1 |
| 90 | 5 min | 1 |
| 12 | Infinite | 1 |

* ramprade of 2 °C/s, heated lid set to 105 °C and sample volume set to 40 µl.

Table S2. ddPCR assays parameters.

| ddPCR assays | Primers | DNA templates concentration | Annealing temperature (°C) | Manual threshold setting |
| --- | --- | --- | --- | --- |
| 16S | Bakt_341F (5’-CCTACGGGNGGCWGCAG-3′)  Bakt_805R (5’-GACTACHVGGGTATCTAATCC-3′) | 2 ∙ 10^-3^ | 51.0 | 5427-7780 |
| ITS | ITS1F (5’-CTTGGTCATTTAGAGGAAGTAA-3′)  58A2R (5’-CTGCGTTCTTCATCGAT-3′) | 2 ∙ 10^-2^ | 53.4 | 7095-13045 |
| *hhyL* | NiFe-244f (5’-GGGATCTGCGGGGACAACCA-3′)  NiFe-568r (5’-TCTCCCGGGTGTAGCGGCTC-3′) | 2 ∙ 10^-2^ | 63.0 | 6000-6980 |

* ramprade of 2 °C/s, heated lid set to 105 °C and sample volume set to 40 µl. DNA template concentration refer to the dilution factor of the extracted DNA samples.


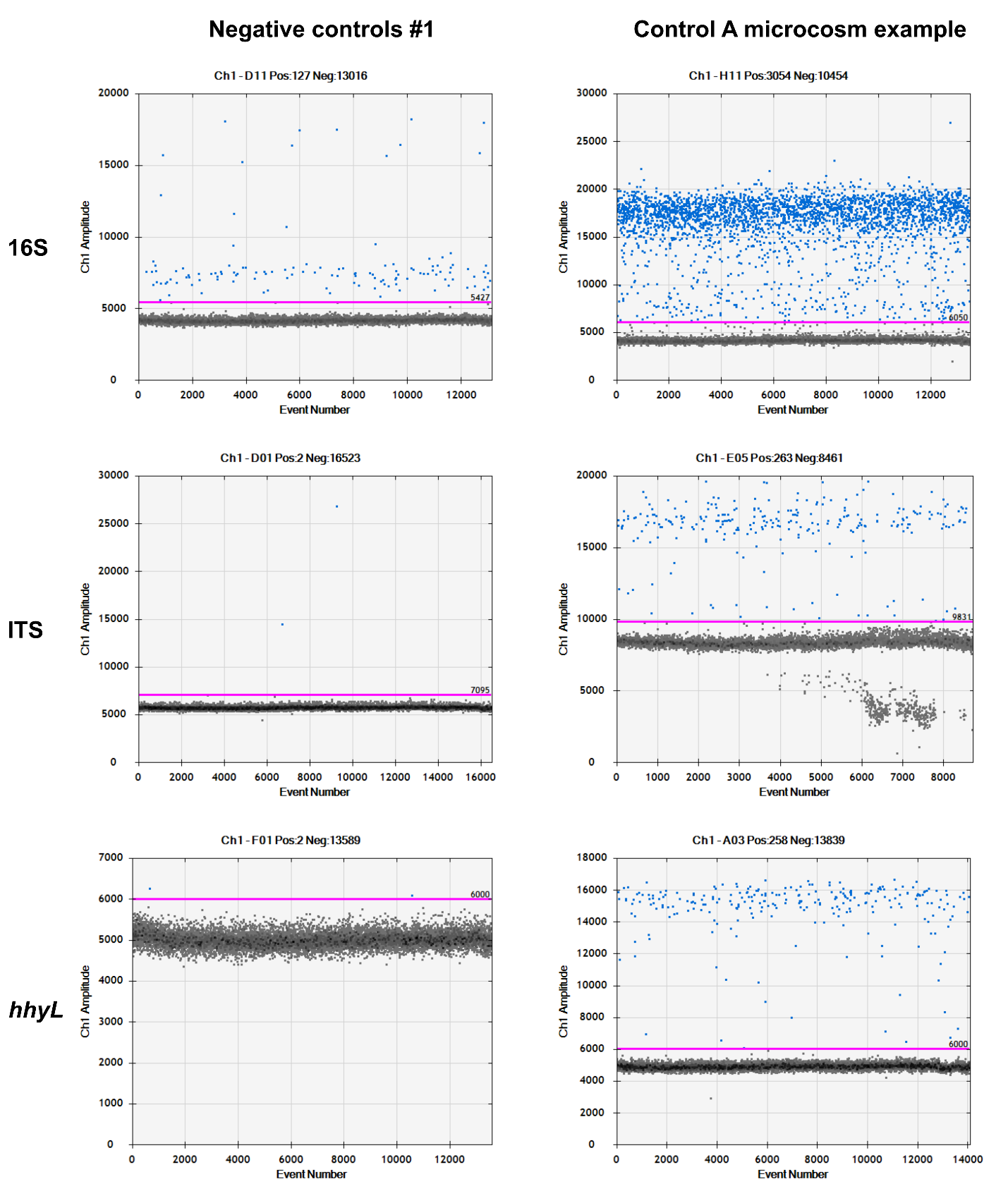

Figure S1. ddPCR graphical display of 1D amplitude of the first negative control 1 and the microcosm Control treatment A with their threshold setting for each ddPCR assays.


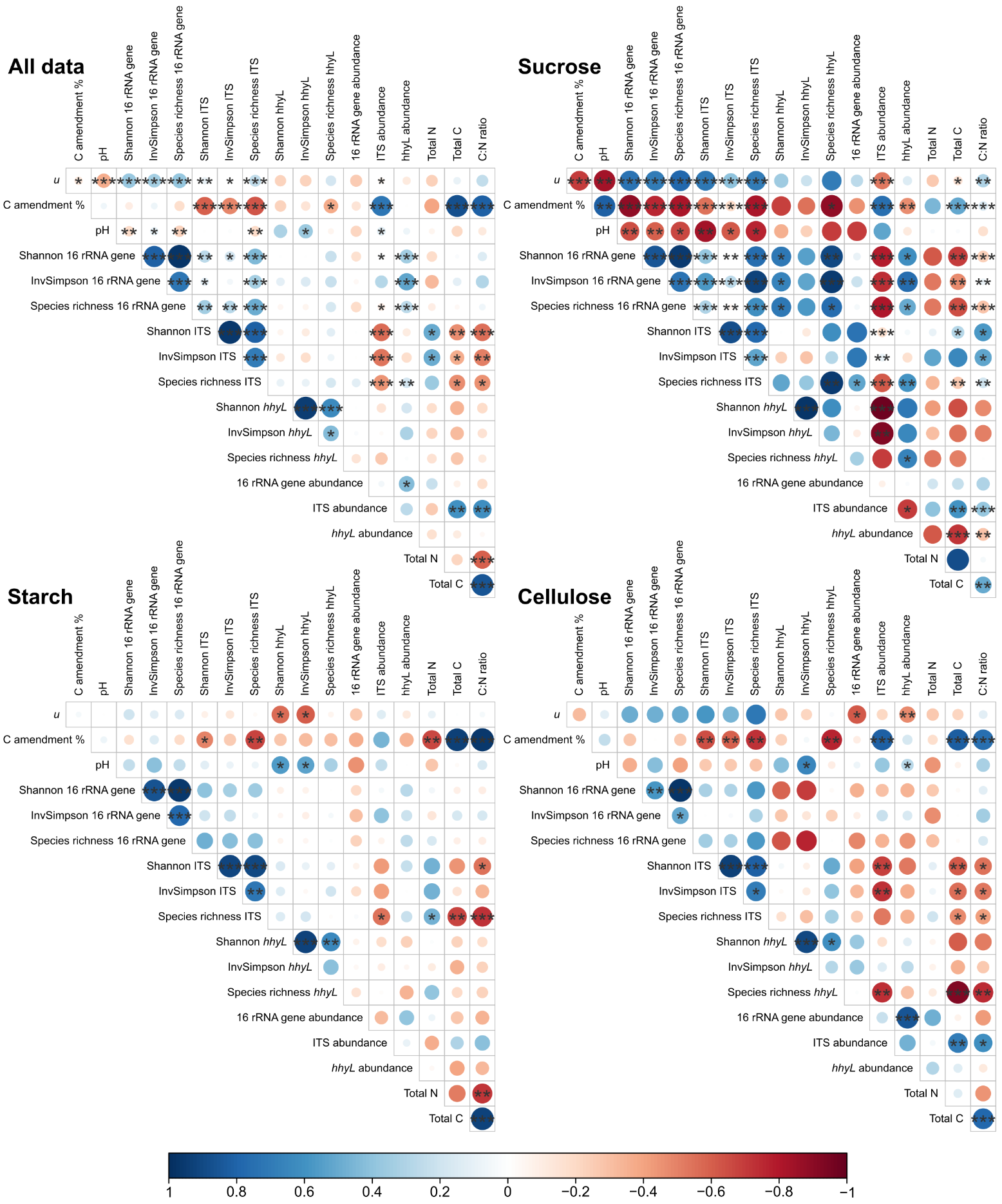

Figure S2. Correlations between the quantitative variables for the complete dataset (All data) and the three subset carbon source amendments. *u*: H_2_ oxidation rate in nmol_H2_ g_dw_^-1^ h^-1^. Colors represent the correlation coefficient, which ranges from 1 to -1. P-values are indicated with asterisks as follows: * for p < 0.05, ** for p < 0.01, and *** for p < 0.001.
